# The Challenge for a European Network of Biobanks for Rare Diseases Taken up by RD-Connect

**DOI:** 10.1159/000358492

**Published:** 2015-03-16

**Authors:** Lucia Monaco, Marco Crimi, Chiuhui Mary Wang

**Affiliations:** Fondazione Telethon, Milan, Italy

**Keywords:** Biobanks, Rare diseases, European network

## Abstract

Access to biological materials is a key prerequisite for scientific research in any medical field and in particular for research into rare diseases (RDs), for which obtaining high-quality samples and the related clinical data remains a major hurdle. RD biobanks play a pivotal role in making such materials and data available to the scientific community. In order to increase the effectiveness of RD biobanks, three major challenges need to be met: maximise access to rare biological samples stored in RD biobanks spread globally by the international scientific community, promote networking among such biobanks to share quality standards and procedures and allow collaboration with RD registries and databases, and finally adopt an efficient management model compliant with legal and ethical issues and ensuring biobank sustainability. The European program RD-Connect, funded under the FP7 program, addresses all of these issues through an articulated action plan aimed at building a network of European RD biobanks. Ultimately, RD-Connect will offer access to precious, quality-controlled biological samples from RD patients through an online, searchable, dynamic catalogue in the context of an integrated platform that links RD patient registries to biobanks and to clinical bioinformatics data for RD research.

## Rare Disease Research: Why Biobanks Matter

Rare diseases (RDs) represent a health care and research challenge in Europe and across the world. Although individually each of the 5,000-8,000 RDs affects a limited proportion of individuals, it is estimated that 6-8% of the population suffers from an RD [1]. Given their rarity, RDs are orphan diseases, being neglected by public and private investments in research and lacking effective treatments [2].

Most RDs are caused by a hereditary genetic defect and, as such, are prone to direct investigation of the pathophysiologic mechanism once the genetic cause has been identified. Fundamental research on rare genetic diseases may disclose general biological pathways and identify therapeutic approaches that will benefit not only patients affected by the specific disease under investigation, but also by common disorders.

Access to high-quality human biological materials and the associated clinical data is a key prerequisite for medical-scientific research on RDs. RD biobanks serve this purpose by collecting biospecimens linked to clinical data from patients affected by RDs and their relatives, and by organising these samples in a systematic way for their distribution to scientists engaged in research on RDs [3].

As such, they offer remarkable research opportunities for the development of diagnostic tools and biomarkers, as indispensable starting points for the study of the disease mechanism eventually leading to the identification of therapeutic targets, and finally for testing therapeutic approaches.

In this paper, the challenges to be addressed in dealing with RD biobanks will be described, along with the potential solutions envisaged within the newly started RD-Connect program funded by the European Commission. The program aims at optimising access to RD biomaterials, in the overall context of a technological platform that comprises patient registries and ‘-omics’ technologies in support of RD research in Europe.

## RD-Biobanks: The European Scenario

Although several biobanks exist in Europe that specifically focus on RDs, or include at least some RD samples in their catalogues, it is not easy to build a comprehensive picture of the European scenario of RD biobanking. Currently, the portal for RDs and orphan drugs, Orphanet, lists 138 biobanks in European countries [4]. Moreover, relevant initiatives on RDs, including RD biobanks, have been supported by European-funding bodies. The European Commission has been supporting research into RDs since the early 1990s under the EU Framework Programmes for Research and Technological Development (FPs) [5]. In particular, specific funding initiatives have been dedicated to biobanking activities.

Specifically, the EuroBioBank (EBB, www.eurobiobank.org/), launched under FP5 and subsequently supported through the FP6 program TREAT-NMD, was the first network of RD biobanks to operate in Europe, and now includes 22 biobanks from 11 countries (9 European) [6]. Moreover, the Biobanking and Biomolecular Resources Research Infrastructure (BBMRI) launched under FP7, one of the largest infrastructure projects in Europe, currently has 54 members and 225 associated partners from 30 countries, and has now developed into a European Research Infrastructure Consortium (ERIC, www.bbmri-eric.eu).

Other funding bodies have also devoted support to biobanking. Among these, the Fondazione Telethon (http://www.telethon.it/en), an Italian charity focused on genetic diseases and a member of the International Rare Diseases Research Consortium (IRDiRC, http://www.irdirc.org/), has been supporting genetic biobanks since 1993 and created, manages and supports the Telethon Network of Genetic Biobanks (TNGB, www.telethon.it/en/scientists/biobanks), the first such activity in Italy, which coordinates 10 biobanks across the country. Of note, all Italian members of the EBB are part of the Fondazione Telethon network. In addition, the Fondazione Telethon has been in charge of the EBB management and financial support since 2012. Collectively, the EBB and Fondazione Telethon biobanks store more than 130,000 samples from more than 700 RDs, largely of genetic origin. Every year, approximately 13,000 of these samples are distributed worldwide, contributing to many highly productive scientific projects, as proven by hundreds of scientific papers published so far. Of note, the TNGB has developed an online searchable catalogue that provides a single entry point for sample requests to all of its biobanks, providing user-friendly access to the samples, linked to a streamlined processing workflow of sample requests [7].

Besides providing valuable biological material to the RD scientific community, both the EBB and TNGB have developed standard operating procedures, patient informed consent forms, and policy and ethical guidelines that have been adopted and implemented by all members. Direct involvement of patient advocacy groups in biobanking initiatives has been encouraged and implemented through dedicated agreements developed within the TNGB, entailing direct donation of biological samples from patients to the relevant biobank.

## RD Biobanks: The Challenges of Rarity

In order to gain maximum impact on scientific research, RD biobanks face some major challenges. The primary challenge is to allow effective access to biological samples and data stored in biobanks [8]. Typically, RD biobanks operate in conjunction with a clinical reference centre for one or more RDs. Therefore, each one reflects the specific clinical competence of the referring centre and may include samples from just one or a limited number of diseases, or a few samples from several different diseases. Making all of these valuable samples equally accessible to the scientific community is of paramount importance not only to promote RD research, but also to fully justify the effort and investment required to create and maintain the biobank. A biobank that does not distribute samples is not fulfilling its primary objective and potentially delays scientific progress and benefit for patients.

Another challenge is that RD biobanks need to be connected within networks that ensure uniformly high quality levels of both biomaterials and associated clinical data, apply harmonised operational procedures, reduce redundancies, optimise investments, and facilitate exchanges of expertise and competences [9]. Moreover, biobanks operating in a network will gain in visibility through a common Web portal that will facilitate access to the biological materials. In general, a biobank operating in isolation has limited effectiveness and outreach. This is particularly true in the field of RDs where, due to the scarcity of biological material, different biobanks may need to be accessed in order to reach significant numbers of samples needed for the study. In these cases, searching for samples through a centralised access point will make this task easier. Furthermore, for RD biobanking, close collaboration with registries and databases, as well as natural history studies and clinical trials, is of particular importance. Therefore, connecting biobank networks to patient registries and databases will further potentiate the effectiveness of the services provided, in line with IRDiRC's recommendations [10].

A third challenge is that high-quality and sustainable operation of the infrastructure is required, in accordance with streamlined procedures that comply with legal and ethical requirements, adopting shared quality standards, providing suitable training to both scientists and technical staff involved in the biobank, and implementing sustainability measures ensuring biobank viability [11]. A biobank lacking a robust and quality-based management model is inefficient and potentially dangerous.

These challenges are all the more cogent in the case of RDs due to the paucity of patients, often entailing the establishment of international collaborations, the need to obtain samples from different sources, the need for validated and standardised diagnostic approaches to identify the disease, the frequent lack of molecular data associated to the samples, and the scarce visibility of minor biobanks. All of these factors make RD biobanks unique and the biological specimens stored therein a precious resource for investigators.

## Facing the Challenges: The RD-Connect Program

Most recently, the FP7 program RD-Connect entitled ‘An Integrated Platform Connecting Databases, Registries, Biobanks and Clinical Bioinformatics for Rare Disease Research’ (www.rd-connect.eu) was launched with the objective of providing ‘centralised access to reference-omics profiles of diseases, based on standardised and validated data and sample collection, and on the integration of databases and bio-banks’ [12]. The 6-year program started on November 1, 2012.

RD-Connect meets the goals of IRDiRC and will contribute towards reaching its ambitious objectives of developing 200 new RD therapies and diagnostic tools for most RDs by the year 2020.

Biobanks represent one of the three pillars of RD-Connect, whose primary objective is to connect research data with clinical information and biological samples related to RDs, allowing full access to and exploitation of such information and biological materials, and subsequently maximising research outcomes to the benefit of RD patients.

Fondazione Telethon coordinates RD-Connect's activities on RD biomaterial sharing and does not only count on the expertise built within the EBB and TNGB, but also, to reach the maximum possible European breadth, it relies on the participation of BBMRI. As previously mentioned, the European Commission funded both the EBB and the preparatory phase of BBMRI; their involvement in RD-Connect does therefore leverage the outcomes of such an investment by exploitation of the expertise, tools and guidelines developed within these programs.

Moreover, in line with the goals of the IRDiRC initiative, global outreach of the biobanking work package is ensured by participation of relevant international partners such as the Office of Rare Diseases Research (ORDR) of the US National Institutes of Health, which created the Rare Disease-HUB (RD-HUB; biospecimens.ordr.info.nih.gov/), a database for biospecimens/biorepositories that serves as a portal to identify and locate RD specimens [13], and with the Australian Office of Population Health Genomics (OHPG, www.genomics.health.wa.gov.au/home/), which developed principles and guidelines for the governance and management of biobanks in Western Australia [14].

## Biomaterial Sharing: RD-Connect's Coordinated Plan

**Fig. 1:**
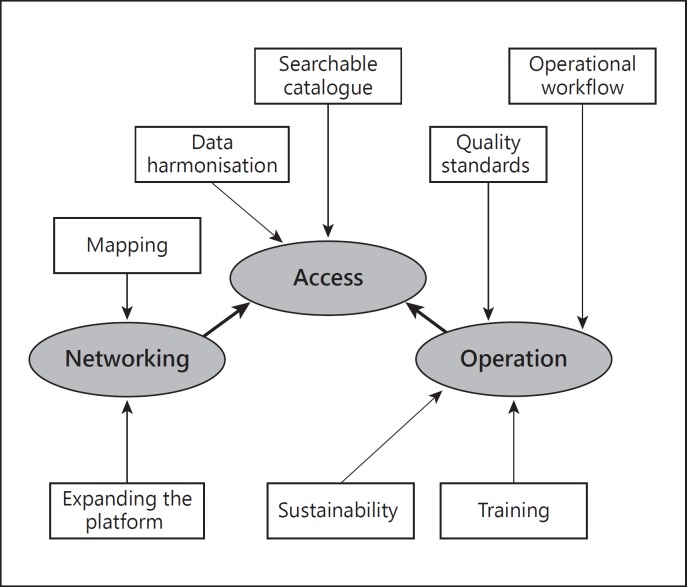
The challenges (ovals) posed by RD biobanking, as addressed by the RD-Connect program through specific actions (rectangles).

In RD-Connect, we address the challenges of RD biomaterial sharing in a comprehensive way through a coordinated plan devising specific actions that tackle each critical point outlined above. Providing access to valuable biological samples through a simple and reliable process will be key to success. To this end, RD-Connect has the ambition to create an online searchable catalogue of biological samples related to RDs, a powerful tool that will serve scientists in Europe and beyond. A new online interface for an RD biomaterial catalogue, including primary cells, tissue, DNA, serum, RNA and human-induced pluripotent stem cell lines will be developed to enable -omics research. Moreover, to facilitate such processes, a streamlined workflow for optimal collection, storage and dissemination of biomaterials will also be implemented. The specific actions devised to meet the three challenges identified above are described below and illustrated in figure 1.

### Map Current Status of RD Biobanks

Building a map of RD biobanks is the essential starting point for the construction of a RD-biobank network. Different surveys have been conducted in an attempt to map existing RD biobanks in Europe and beyond [15,16], but to our knowledge no dynamic and easily manageable database for RD biobanks has been developed. Such a database will include European biobank networks already established with EU support (EBB and BBMRI-ERIC) and existing networks, such as the TNGB, and will provide reference to large networks outside Europe (in the USA and Australia). The survey will build on previous surveys of the European biobanking landscape performed in BBMRI and will include information on governance, available biological samples and data, data management, quality management, and access procedures [17]. Within RD-Connect, this activity will be run in parallel with a similar mapping exercise regarding RD registries.

### Develop and Implement a Shared Dataset for RD Biobanks and Implement a Standardised Coding System Linked to a Unique Patient ID

Producing a set of minimum common data elements is key to the creation of a biobank biological sample catalogue that will ultimately facilitate access to RD biospecimens. Definition of a shared dataset for RD biobanks will leverage existing initiatives in Europe, the USA and Australia and will be developed in close collaboration with similar efforts being undertaken for databases and registries within the RD-Connect program, in order to allow ultimate cross-referencing among biobanks, registries and -omics databases.

### Develop a Centralised Searchable Biological Sample Catalogue

An online, dynamic, searchable catalogue of samples collected in RD biobanks is a fundamental tool to allow access to samples to be employed in -omics studies. The catalogue will federate existing catalogues identified through the mapping activity and employ standardised datasets and coding systems, as described above. Activities will build on the BBMRI experience [18]. The ‘core’ catalogue will include samples from established biobank networks, such as the TNGB and EBB. The catalogue will be progressively expanded to include other biobanks relevant to RD-focused projects. Such expansion will require implementation of a validation process on data quality of the catalogue (based on quality standards developed in Task 5). Semantic technologies will be used for enhanced searchability and identification of relevant biobanks, biobank-user, researchers, patients and samples.

### Set and Implement Quality Standards for RD Biobanks

Harmonisation of quality standards is essential for optimal operation of biobanks within a network. Such standards include standard operating procedures for sample and data collection, work-up, storage and distribution, as well as legal and ethical forms for sample collection and distribution. Leveraging the wide experience developed within the BBMRI and the EU-funded SPIDIA project (http://www.spidia.eu/), which is developing the scientific basis for evidence-based European standards and norms for sample pre-analytics, a shared set of standards will be defined and adopted/implemented by biobanks within the RD-Connect platform. A survey across participating organisations in Europe, USA and Australia will be run in order to produce a report of existing guidelines that will serve as the basis for the biobanking quality standard guidelines. Bioethics and legal issues will be developed, as well as quality standards for samples to be employed in -omics studies.

### Incorporate New Biobanks into the Platform through Assessment of Incoming Biobanks and Online Registration System

Following the establishment of the biobank catalogue, entry of new biobanks into the network is envisaged and encouraged. To this end, an assessment procedure will be established. This will entail development of assessment criteria and set-up of a panel for validation of incoming biobanks, with the aim of ensuring adherence to minimal entry conditions and adoption of any standardisation and harmonisation measures needed for inclusion into the platform. An online registration system to upload information and data of the new biobank into the catalogue will be developed.

### Recruit Samples from RD Patients Also through Patient Organisations and Disseminate Samples to -Omics Projects

Although the task of collecting samples and distributing them to researchers must remain the responsibility of individual biobanks, outlining an efficient and effective workflow would facilitate such processes. In particular, dissemination activities among patient advocacy groups should be encouraged and facilitated by providing information, template agreements and advice. Therefore, quality standards regarding collection of samples and associated data, sample preparation and storage, and sample distribution, as previously described, will be incorporated into a workflow meant to facilitate biobank operational activities. Policies regulating criteria of access to the samples will be developed.

### Develop Training Materials and Hold Training Workshops

This activity addresses the need for the development of training materials relevant to the operation of biobanks, in particular for incoming biobanks, and will include training workshops as well. Moreover, although several collections of RD-related biomaterials exist, not all of them belong to bona fide biobanks, i.e. to organised infrastructures performing collection, storage and distribution services according to standardised procedures in compliance with legal and ethical regulations [19]. Through this action, essential information and material for the graduation of collections into fully fledged biobanks will be provided.

### Investigate Sustainability Options for RD Biobank Networking

Financial sustainability of multinational biobank networking initiatives is recognised as a major issue for the successful operation and management of biobanks. In general, the basic sustainability of biobanks strongly depends on the background support from host partners (such as academic hospitals or private companies). Other financial support is usually uncertain, therefore biobanks, whether they belong to a public or private sector, must continuously look for multi-source financing models accounting for public government funds, private venture capitals, not-for-profit charitable funds and collaborations with commercial entities [20]. Although a reasonable cost-recovery model based on a standard accounting system is ethically acceptable, it should be stressed that RD-oriented biobanks should never offer any financial reimbursement to patients as an incentive to donate their biological samples, or sell for profit samples that they have collected. Building on BBMRI experience with its new ERIC infrastructure and exploring global options with US and Australian partners, sustainability options will be studied and developed into a sustainability plan.

## Prospects and Conclusions

The RD biobank platform created within the RD-Connect program will produce two relevant outcomes for RD research: (1) the catalogue of RD biomaterials, a searchable, dynamic tool that will provide direct access to high-quality biospecimens and related data stored in biobanks across Europe, fundamental for research into RDs, and (2) quality standard guidelines and an operational workflow for the optimal conduct of RD biobanks.

Such results will be potentiated and amplified by the interactions with the other RD-Connect components - patient registries and bioinformatics tools - all of them supported by an innovative data informatics management.

It is therefore expected that RD-Connect will have a huge scientific impact, as it will ensure that biological samples donated by RD patients and their linked clinical data will be accessible and available to the scientific community through an internationally coordinated system.

Different types of users - scientists and clinicians from both academic and commercial entities - will be able to use the newly generated analytic tools in compliance with ethical guidelines and with the laws in force. Novel studies on biological samples obtained through the development of the RD-Connect catalogue of biobanks and networking infrastructures will lead to new knowledge on RDs, and new diagnostic tools and therapeutic approaches for the benefit of RD patients and in line with the IRDiRC objectives.

Two years after the start of the RD-Connect program, significant progress has been achieved: a dedicated Web-crawling tool was built and the survey aimed at mapping RD biobanks and networks in Europe is delivered, minimal information standards for sample collections have been defined, and the infrastructures to host these data, as well as the centralized sample catalogue, have been built and are now being tested. In addition, effective collaboration between biobanking initiatives in Europe and abroad has been established, and the biobanking operative workflow has been designed so that all premises point to a productive prosecution of this challenging and promising program.
